# Lakeside Cemeteries in the Sahara: 5000 Years of Holocene Population and Environmental Change

**DOI:** 10.1371/journal.pone.0002995

**Published:** 2008-08-14

**Authors:** Paul C. Sereno, Elena A. A. Garcea, Hélène Jousse, Christopher M. Stojanowski, Jean-François Saliège, Abdoulaye Maga, Oumarou A. Ide, Kelly J. Knudson, Anna Maria Mercuri, Thomas W. Stafford, Thomas G. Kaye, Carlo Giraudi, Isabella Massamba N'siala, Enzo Cocca, Hannah M. Moots, Didier B. Dutheil, Jeffrey P. Stivers

**Affiliations:** 1 Department of Organismal Biology and Anatomy, University of Chicago, Chicago, Illinois, United States of America; 2 Dipartimento di Filologia e Storia, University of Cassino, Cassino, Italy; 3 Naturhistorisches Museum Wien, Wien, Austria; 4 School of Human Evolution and Social Change, Arizona State University, Tempe, Arizona, United States of America; 5 Laboratoire d'Océanographie et du Climat Expérimentations et Approches Numériques, Université Pierre et Marie Curie, Paris, France; 6 Direction de L'Education, Culture, Science et Technologie, Economic Community of West African States Commission, Abuja, Nigeria; 7 Institut des Sciences Humaines, Université de Niamey, Niamey, République du Niger; 8 Dipartimento del Museo di Paleobiologia e dell'Orto Botanico, Università di Modena e Reggio Emilia, Modena, Italy; 9 Stafford Research Laboratories, Inc., Lafayette, Colorado, United States of America; 10 Burke Museum of Natural History, University of Washington, Seattle, Washington, United States of America; 11 Ente per le Nuove Tecnologie, l'Energia e l'Ambiente, Rome, Italy; 12 Dipartimento di Scienze Storiche, Antropologiche e Archeologiche dell'Antichità, University of Rome “La Sapienza,” Rome, Italy; 13 Muséum national d'Histoire naturelle, Paris, France; 14 Federal Way, Washington, United States of America; University of Utah, United States of America

## Abstract

**Background:**

Approximately two hundred human burials were discovered on the edge of a paleolake in Niger that provide a uniquely preserved record of human occupation in the Sahara during the Holocene (∼8000 B.C.E. to the present). Called Gobero, this suite of closely spaced sites chronicles the rapid pace of biosocial change in the southern Sahara in response to severe climatic fluctuation.

**Methodology/Principal Findings:**

Two main occupational phases are identified that correspond with humid intervals in the early and mid-Holocene, based on 78 direct AMS radiocarbon dates on human remains, fauna and artifacts, as well as 9 OSL dates on paleodune sand. The older occupants have craniofacial dimensions that demonstrate similarities with mid-Holocene occupants of the southern Sahara and Late Pleistocene to early Holocene inhabitants of the Maghreb. Their hyperflexed burials compose the earliest cemetery in the Sahara dating to ∼7500 B.C.E. These early occupants abandon the area under arid conditions and, when humid conditions return ∼4600 B.C.E., are replaced by a more gracile people with elaborated grave goods including animal bone and ivory ornaments.

**Conclusions/Significance:**

The principal significance of Gobero lies in its extraordinary human, faunal, and archaeological record, from which we conclude the following:

1. The early Holocene occupants at Gobero (7700–6200 B.C.E.) were largely sedentary hunter-fisher-gatherers with lakeside funerary sites that include the earliest recorded cemetery in the Sahara.
2. Principal components analysis of craniometric variables closely allies the early Holocene occupants at Gobero with a skeletally robust, trans-Saharan assemblage of Late Pleistocene to mid-Holocene human populations from the Maghreb and southern Sahara.
3. Gobero was abandoned during a period of severe aridification possibly as long as one millennium (6200–5200 B.C.E).
4. More gracile humans arrived in the mid-Holocene (5200–2500 B.C.E.) employing a diversified subsistence economy based on clams, fish, and savanna vertebrates as well as some cattle husbandry.
5. Population replacement after a harsh arid hiatus is the most likely explanation for the occupational sequence at Gobero.
6. We are just beginning to understand the anatomical and cultural diversity that existed within the Sahara during the Holocene.

## Introduction

The “greening” and ultimate desiccation of the Sahara rank among the most severe climatic fluctuations during the Holocene [1]. Driven by variation in orbital insolation and magnified by feedback between monsoonal rainfall and vegetation [2], ecosystem succession in the Sahara is well known from many lines of evidence such as pollen spectra [3], paleolake levels [4]–[6], and, most recently, high-resolution paleolake sediment cores [7].

Human adaptation during this period of climate fluctuation is best known in the Eastern Sahara to the west of the Nile valley. This region witnessed continuous occupation from 8500 B.C.E., when hunter-gatherers using a distinctive Epipaleolithic tool kit expanded across open grass savanna habitats, to about 3500 B.C.E, when aridification drove pastoralists from most areas of the desert [8]. Occupational patterns in low-lying regions elsewhere in the Sahara most closely resemble the Eastern Sahara during the early Holocene (∼8000–7000 B.C.E.), when pottery-producing hunter-fisher-gatherers resided beside paleolakes, utilizing a tool kit including microliths and harpoon points and fish hooks of bone [9]–[11]. By the mid-Holocene, occupational histories diverge in the Central and Western Sahara [12] as a result of distinctive local humid-arid cycles [13]–[15], diversified economies and lifestyles tied to ephemeral paleolakes [10], upland refugia [16] and rivers [17], and marked variation among the human populations themselves [10], [18], [19]. Despite increasing knowledge regarding occupational succession in the Sahara from early to late Holocene [8], [10], [11], [16], [17], that record is based on individual sites that typically preserve short intervals of occupation, include few if any intact burials, and rely largely on indirect dating of human remains and artifacts [20].

**Figure 1:**
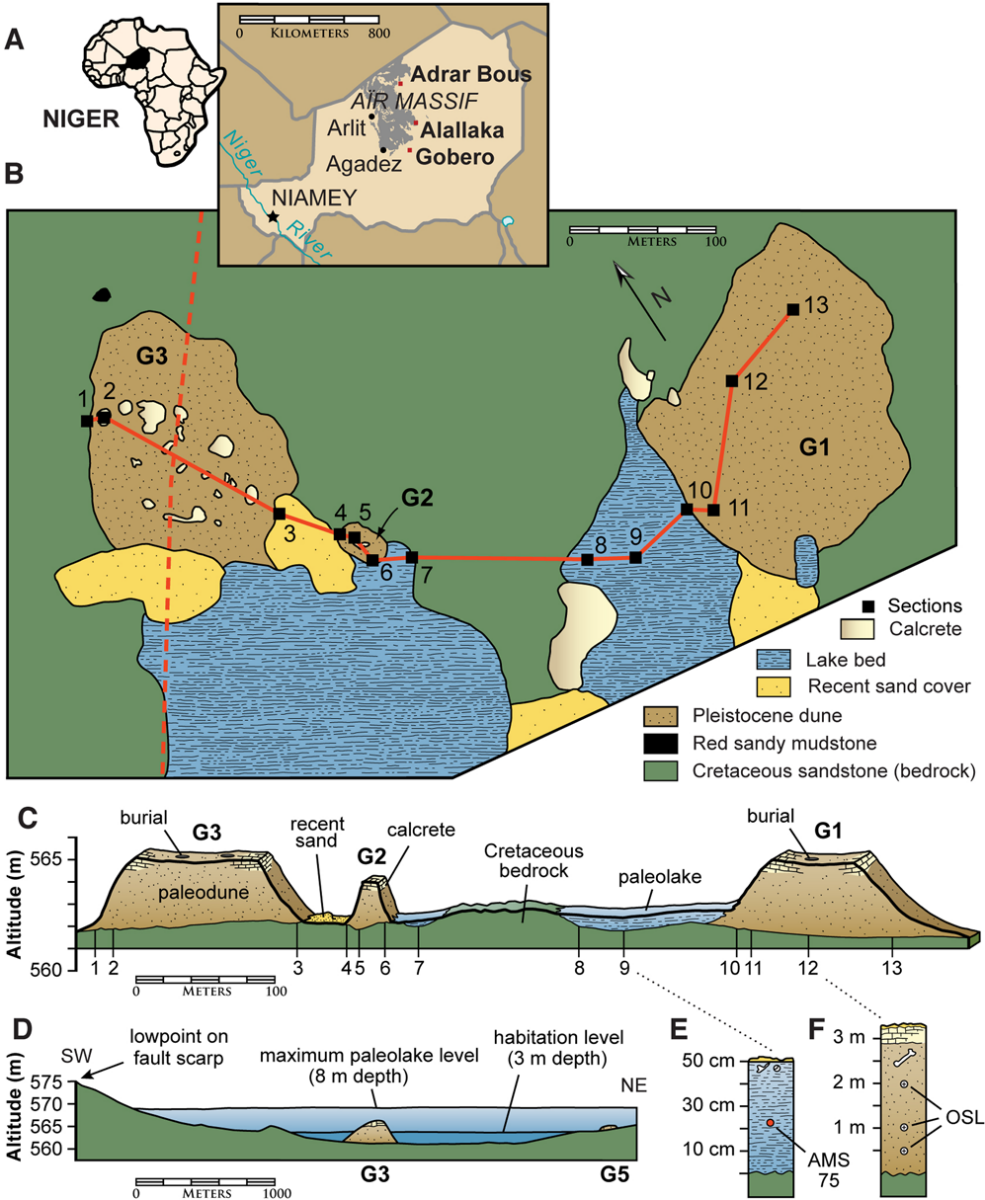
Location maps and geologic section across principal sites at Gobero. (A)-Map showing location of the Holocene archaeological site Gobero and the Holocene felsite quarry Alallaka on the border of the Aïr massif. (B)-Geologic map of main paleodune cemetery sites (G1-3) showing transect line connecting 13 geologic sections (see C, E, F) and a portion of a topographic transect (dashed line; see D). (C)-Stratigraphic profile across sites G1-3 based on 13 sections showing the Cretaceous peneplain of the Elrhaz Formation, the Late Pleistocene to early Holocene paleodune deposit, the early to mid-Holocene paleolake deposit, and Recent sand cover (15 times vertical exaggeration). (D)-Topographic transect (dashed line in B) between site G5 and a spillway on the Mazelet fault scarp located 1.3 km to the south (30 times vertical exaggeration). Habitation (3 m) and maximum (8 m) paleolake levels are shown, the latter resulting in inundation of archaeological sites G1-5. (E)-Stratigraphic section of paleolake deposit between sites G1 and G2 with fossiliferous zone limited to the uppermost 5 cm and location of sediment sample for ^14^C AMS date 75 (Table 2). (F)-Stratigraphic section of paleodune deposit at site G1 showing human skeletons limited to the uppermost 1 m and the location of three OSL samples (Table 1, dates 2–4). *Abbreviations*: *AMS 75*, ^14^C AMS date 75; *OSL*, optically stimulated luminescence.

**Figure 2:**
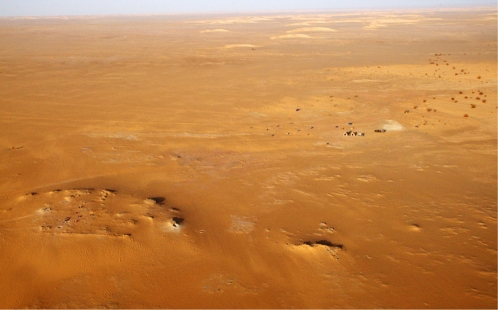
Aerial view of Gobero sites. Aerial view (facing north) of a portion of the Gobero site complex, first discovered in 2000, showing the raised paleodune sites G3 (bottom left, oval) and G2 (bottom right, ridge) situated on the peneplain of the Early Cretaceous (Aptian-Albian) Elrhaz Formation. The 2006 Expedition campsite (middle right) and a Recent barcan dune field are seen in the distance. The prominent edges of the paleodune sites are composed of calcrete (calcite-cemented aeolian sand). An excavation team is present on site G3. Near its right (east) margin, the pit for geologic section 3 is visible (see Figure 1B).

We report here on a new site complex called Gobero located at the western tip of the hyperarid Ténéré Desert in the southern Sahara in Niger (Figures 1A, 2). Approximately 200 burials ranging in age over five millennia are present in the upper level of several paleodunes that are situated adjacent to a paleolake deposit. Gobero preserves the earliest and largest Holocene cemetery in the Sahara, opening a new window on the funerary practices, distinctive skeletal anatomy, health and diet of early Holocene hunter-fisher-gatherers, who expanded into the Sahara when climatic conditions were favorable. The site complex also preserves numerous mid-Holocene burials, some indicating funerary rituals with grave inclusions. Associated middens and an exceptional faunal and pollen record provide a chronicle of episodic human occupation in the Sahara under conditions of severe climatic change.

**Table 1:** Optically stimulated luminescence (OSL) dates for paleodune sand.

No.	Sample Level	Notes	Lab-Date-Number	OSL Age	
				BP	BCE
***Site G1***					
**1**	mid level	1.5 m down from calcrete	UG-2006-391	10,100±700 BP	8100±700 BCE
**2**	upper level (section 12)	1.0 m down from calcrete; burial level; deep control trench	UG-2007-442	10,500±800 BP	8500±800 BCE
**3**	mid level (section 12)	2.0 m down from calcrete; deep control trench	UG-2007-443	16,500±1800 BP	14,500±1800 BCE
**4**	lower level (section 12)	2.5 m down from calcrete; deep control trench	UG-2007-445	15,000±1400 BP	13,000±1400 BCE
***Site G2***					
**5**	upper level	1.0 m down from calcrete	UG-2007-444	8700±800 BP	6700±800 BCE
***Site G3***					
**6**	upper level	0.35 m down from calcrete	UG-2006-393	8200±600 BP	6200±600 BCE
**7**	upper level	1.0 m down from calcrete	UG-2007-446	10,800±1200 BP	8800±1200 BCE
**8**	upper level	0.75 m down from calcrete; thickness of dune 2.0–3.0 m	UG-2006-394	11,100±1200 BP	9100±1200 BCE
***Site G4***					
**9**	G4 upper level	1.0 m down from calcrete	UG-2007-448	10,500±1200 BP	8500±1200 BCE

Nine OSL dates on paleodune sand samples taken at varying depth from sites G1-4 at Gobero. OSL samples 2–4 are from a 3.0 m section on the north edge of site G1 (Figure 1F). *Abbreviations*: *BCE*, BP dates minus 2000 years to approximate BCE age; *BP*, before present; *UG*, Luminescence Dating Laboratory, University of Georgia.

**Table 2:** Ages and associated data for 78 radiocarbon ^14^C AMS dates, which are shown graphically in Figure 3 (bottom to top).

Date No.	Sample Type	Lab No.	∂^13^C ‰ (PDB)	^14^C Age (conventional)	Cal BC (95%) (midpoint)	Specimen	Notes
***Human Bone & Enamel***							
**1**	enamel	P-587	−4.16	**8640±40 BP**	7730–7580 (7655 cal BC)	**G3B7**	Subadult in vertical burial (dark-stained bone)
**2**	enamel	P-590	−4.85	**8620±40 BP**	7680–7580 (7630 cal BC)	**G3B17B**	Double juvenile burial (4- & 5-year olds); 5-year old (dark-stained bone)
**3**	enamel	P-542	−3.35	**8570±40 BP**	7600–7550 (7575 cal BC)	**G3B9**	Adult male (dark-stained bone)
**4**	femur (internal)	P-583	−5.35	**8470±40 BP**	7580–7490 (7535 cal BC)	**G3B8**	Adult male, tightly hyperflexed, supine; sherd with dotted wavy line decoration under skeleton (dark-stained bone)
**5**	enamel	P-584	−5.08	**8420±40 BP**	7570–7460 (7515 cal BC)	″	″
**6**	femur (surface)	P-582	−5.50	**8220±40 BP**	7350–7080 (7215 cal BC)	″	″
**7**	enamel	P-546	−5.93	**8330±40 BP**	7510–7310 (7410 cal BC)	**G3B23**	Adult female (dark-stained bone)
**8**	enamel	P-544	−5.37	**7390±40 BP**	6380–6210 (6295 cal BC)	**G3B28**	Adult (dark-stained bone)
**9**	enamel	P-595	−4.60	**5940±40 BP**	4930–4720 (4825 cal BC)	**G1B11**	Adult male with mud turtle carapace *(Pelusios)* under skeleton (intermediate-stained bone)
**10**	enamel	P-541	−3.81	**5620±40 BP**	4530–4360 (4445 cal BC)	″	″
**11**	femur	P-581	−3.48	**5570±40 BP**	4470–4340 (4405 cal BC)	″	″
**12**	enamel	P-634	−5.02	**5140±40 BP**	4030–3810 (3920 cal BC)	**G2B1**	Adult male (dark-stained bone)
**13**	enamel	P-633	−4.60	**4990±40 BP**	3940–3660 (3800 cal BC)	**G5B2**	Adult male (dark-stained bone)
**14**	enamel	P-586	−7.05	**5180±40 BP**	4040–3950 (3995 cal BC)	**G3B6**	Adult female
**15**	enamel	P-540	−7.60	**4990±40 BP**	3940–3660 (3800 cal BC)	**G3B5**	Adult male
**16**	enamel	P-585	−1.90	**4910±40 BP**	3770–3640 (3705 cal BC)	**G3B3**	Adult male
**17**	enamel	P-545	−6.77	**4860±40 BP**	3700–3540 (3620 cal BC)	**G3B41**	Adult female with necklace
**18**	enamel	P-543	−4.75	**4710±40 BP**	3630–3370 (3500 cal BC)	**G3B36**	Adult male, skull in half ceramic pot; grave goods include croc astragalus, boar tusk
**19**	enamel	P-547	−6.25	**4690±40 BP**	3630–3360 (3495 cal BC)	**G3B24**	Adult near Midden 4
**20**	enamel	P-594	−3.57	**4590±40 BP**	3500–3130 (3315 cal BC)	**G1B8**	Adult female in triple burial
**21**	femur	P-589	−2.63	**4090±40 BP**	2860–2490 (2675 cal BC)	″	″
**22**	enamel	P-588	−5.28	**4380±40 BP**	3100–2900 (3000 cal BC)	**G1B5**	Juvenile male with frontal osteoporosis
**23**	enamel	P-548	−3.95	**4370±40 BP**	3090–2900 (2995 cal BC)	**G1B7**	Adult female
**24**	enamel	P-592	−3.13	**4360±40 BP**	3090–2900 (2995 cal BC)	**G1B6**	Adult female, partial toad skeleton found under skull
**25**	enamel	P-593	−1.76	**4250±40 BP**	2910–2760 (2835 cal BC)	**G1B2**	Juvenile female with upper arm bracelet
***Ceramics***							
**26**	partial pot	P-539	−20.40	**8150±40 BP**	7300–7060 (7180 cal BC)	**GA107**	In situ in lakebed between G1 and G2, packed zigzags (Kiffian)
**27**	sherd	P-535	−14.70	**8060±40 BP**	7080–6840 (6960 cal BC)	**G3-98**	Under skeleton G3B8 (dark-stained bone), dotted wavy line (Kiffian)
**28**	sherd	P-538	−17.56	**7570±40 BP**	6470–6390 (6430 cal BC)	**G3-97**	In ribcage of skeleton G3B3 (light-colored bone), 20 cm down, packed zigzag (Kiffian)
**29**	sherd	P-611	−16.50	**6170±40 BP**	5220–5000 (5110 cal BC)	**G1-135**	In burial G1B16 (light-colored bone), level 1, packed zigzag (Kiffian)
**30**	sherd	P-533	−17.40	**5170±40 BP**	4040–3940 (3990 cal BC)	**G3-96**	In burial G3B36 (intermediate-stained bone), level 2, alternating pivoting stamp (Tenerean)
**31**	partial pot	P-534	−15.40	**5130±40 BP**	3990–3800 (3895 cal BC)	**G3-94**	In burial G3B36 (intermediate-stained bone), level 2, alternating pivoting stamp (Tenerean)
**32**	sherd	P-612	−24.27	**5020±40 BP**	3950–3700 (3825 cal BC)	**G3-90**	In burial G3B16 (dark-stained bone), level 1, spaced zigzag (Kiffian)
**33**	sherd	P-536	−17.26	**5010±40 BP**	3940–3700 (3820 cal BC)	**G3-95**	in burial G3B36 (intermediate-stained bone), level 2, packed zigzag (Kiffian)
**34**	sherd	P-613	−21.59	**4950±40 Bp**	3800–3650 (3725 cal BC)	**G3-91**	In burial G3B24 (light-colored bone), level 1, spaced zigzag (Kiffian)
**35**	partial pot	P-615	−15.75	**3640±40 BP**	2130–1900 (2015 cal BC)	**G8-3**	In situ in lakebed at G8, undecorated (Late Tenerean)
**36**	partial pot	P-616	−13.94	**2240±40 BP**	390–200 (295 cal BC)	**GA17**	In situ over charcoal layer on red sandstone west of G3, undecorated (Late Tenerean)
***Charcoals***							
**37**	charcoal	UCIAMS 34745	—	**3555±25 BP**	1980–1770 (1875 cal BC)	**G1B3**	Particle in G1B3 burial (weak acid-base treatment)
**38**	charcoal	UCIAMS 35600	−15.70	**2345±25 BP**	510–370 (440 cal BC)	**GA17a,b**	Charcoal from a hearth in situ directly under pot GA17, GA17a (date 38) is acid-base-HNO_3_-treated, GA17b (date 39) is (humic acids from pretreatment)
**39**	charcoal	UCIAMS 35601	−14.30	**2205±25 BP**	370–190 (280 cal BC)		
***Middens***							
**40**	bone	P-682	−2.79	**7700±40 BP**	6620–6460 (6540 cal BC)	**86N–140E**	Refuse area 5, *Redunca redunca* phalanx (dark-stained bone)
**41**	bone	P-683	−5.21	**7480±40 BP**	6430–6240 (6335 cal BC)	**86N–140E**	Refuse area 5, *Lates niloticus* vertebra, top 5 cm
**42**	clam	UCIAMS 35940	−3.50	**5665±20 BP**	4545–4455 (4500 cal BC)	**—**	Midden 4 (HCl-etched nacreous shell), near G3B24
**43**	clam	UCIAMS 35941	−0.80	**5535±20 BP**	4450–4330 (4390 cal BC)	**—**	Midden 4 (chalky exterior shell); midden near G3B24
**44**	sherd	P-529	−17.58	**5240±40 BP**	4230–3970 (4100 cal BC)	**G1-132**	Midden 1, level 1, alternating pivoting stamp (Tenerean)
**45**	sherd	P-531	−13.77	**5090±40 BP**	3970–3790 (3880 cal BC)	**G1-131**	Midden 1, level 1, packed zigzag (Kiffian)
**46**	sherd	P-530	−13.78	**5030±40 BP**	3950–3710 (3830 cal BC)	**G1-133**	Midden 1, level 2, packed zigzag (Kiffian)
**47**	clam	UCIAMS 35944	−3.90	**5065±20 BP**	3950–3790 (3870 cal BC)	**Clam A**	Midden 1 (chalky exterior shell)
**48**	clam	UCIAMS 35946	−0.40	**5040±15 BP**	3950–3780 (3865 cal BC)	**Clam B**	Midden 1 (HCl-leached hinge, nacreous shell)
**49**	clam	UCIAMS 35945	−1.20	**5005±15 BP**	3910–3710 (3810 cal BC)	**Clam C**	Midden 1 (150 µm thick section, innermost calcite)
**50**	bone	P-549	0.45	**4210±40 BP**	2900–2670 (2785 cal BC)	**G1-136**	Midden 1, level 2, *Bos taurus* femur found in situ (107N, 132E) on G1; proximal femur with epiphysis
**51**	sherd	P-532	−15.77	**4600±40 BP**	3500–3190 (3345cal BC)	**G1-134**	Midden 2, level 1, undecorated (? tradition)
**52**	sherd	P-537	−13.95	**4530±40 BP**	3360–3090 (3345 cal BC)	**G1-135**	Midden 2, level 1, packed zigzag (Kiffian)
**53**	bone	UCIAMS 35598	−6.40	**4445±25 BP**	3330–3010 (3170 cal BC)	**—**	Midden 2 (humic acids from “decalcification supernatant silt-charcoal”)
**54**	bone	UCIAMS 35599	−8.90	**4445±25 BP**	3330–3010 (3170 cal BC)	**—**	Midden 2 (humic acids from decalcified burnt bone)
***Fauna***							
**55**	bone	P-689	−1.13	**8820±40 BP**	8200–7740 (7970 cal BC)	**G1-49**	Bovid astragalus on top of G1, deflated (dark-stained bone)
**56**	bone	P-687	1.83	**7810±40 BP**	6690–6580 (6635 cal BC)	**GF22**	*Alcelaphus buselaphus* metatarsus midshaft section (2 cm) in situ in lakebed G7 (dark-stained bone)
**57**	bone	P-688	−0.15	**7720±40 BP**	6640–6470 (6555 cal BC)	**—**	*Lates niloticus* vertebra, lakebed G6, deflated
**58**	bone	P-691	0.61	**7210±40 BP**	6560–6430 (6495 cal BC)	**GF111**	*Lates niloticus* vertebra, 5 cm diameter, in situ between G1–G2, (dark-stained bone)
**59**	enamel	P-607	−0.53	**4990±40 BP**	3940–3660 (3800 cal BC)	**GF10**	*Bos taurus* molar
**60**	bone	P-608	1.15	**4930±40 BP**	3790–3640 (3715 cal BC)		*Bos taurus* mandible (dark-stained bone)
**61**	bone	P-685	−1.39	**4130±40 BP**	2880–2570 (2725 cal BC)	**GF75**	*Oryx dammah* petrosal, in situ in lakebed south of G3
**62**	bone	P-690	1.75	**4050±40 BP**	2840–2480 (2660 cal BC)	**GF13**	*Hippotragus equinus* astragalus, lakebed, deflated
**63**	bone	P-686	−4.81	**3910±40 BP**	2480–2290 (2385 cal BC)	**S21/43/SR7290**	*Crocodylus niloticus* scute in lakebed, deflated
***Artifacts***							
**64**	bone harpoon point	P-694	−9.00	**7690±50 BP**	6590–6470 (6530 cal BC)	**G3-86a**	Harpoon, in situ in Refuse area 5
**65**	bone harpoon point	P-695	−0.90	**7850±50 BP**	6770–6600 (6685 cal BC)	**G3-86b**	Harpoon, in situ in Refuse area 5
**66**	bone harpoon point	P-692	−0.20	**5500±40 BP**	4450–4270 (4360 cal BC)	**GA59**	Harpoon, in situ in G6 lakebed
**67**	ostrich eggshell bead	CEDAD	−6.10	**5348±55 BP**	4330–4040 (4185 cal BC)	**G3-1a**	eggshell bead associated with burial G3B4 and encrusted with calcrete
**68**	bone harpoon point	P-693	3.60	**5130±50 BP**	3990–3800 (3895 cal BC)	**GA41**	Harpoon, in situ in G6 lakebed
***Sediments & Molluscs***							
**69**	snail	UCIAMS 35937	5.00	**6650±20 BP**	5625–5535 (5580 cal BC)	**GF80**	G1-2 lakebed (HCl-leached gastropods, *Melanoides*)
**70**	clam	UCIAMS 35597	−15.50	**5745±30 BP**	4690–4500 (4595 cal BC)	**—**	G1-2 lakebed clam, black particulate carbon from sediment filling shell interior
**71**	clam	UCIAMS 35596	−14.70	**5505±25 BP**	4450–4270 (4360 cal BC)	**—**	G1-2 lakebed clam, humic acids from sediment filling shell interior
**72**	clam	UCIAMS 35942	0.70	**4955±20 BP**	3790–3660 (3725 cal BC)	**—**	G1-2 lakebed clam, chalky exterior, outer shell surface
**73**	clam	UCIAMS 35943	0.80	**4940±20 BP**	3770–3650 (3710 cal BC)	**—**	G1-2 lakebed clam, HCl-etched nacreous hinge
**74**	calcrete	ISGS	−3.50	**5120±30 BP**	3990–3800 (3895 cal BC)	**G3**	G3 calcrete, east side; inorganic C from CO_3_
**75**	lakebed	ISGS	−17.50	**6250±180 BP**	5550–4750 (5150 cal BC)	**G6**	G6 lakebed sediment, 20–25 cm depth; below organic rich layer; low organic C yield
**76**	clam	CEDAD	1.70	**4562±35 BP**	3490–3100 (3295 cal BC)	**G6**	G6 lakebed clam, composite shell (did not isolate organic layer)
**77**	clam	UCIAMS 35939	2.00	**4420±20 BP**	3270–2920 (3095 cal BC)	**G6a,b**	G6 lakebed clam, G6a (date 77) is HCl-leached hinge zone shell, G6b (date 78) is from chalky exterior shell
**78**	clam	UCIAMS 35938	0.80	**4395±20 BP**	3090–2920 (3005 cal BC)		

Dates from the same specimen or feature are grouped together (shaded or unshaded). For some sherds recovered in burials with Kiffian decorative motifs, dates based on plant temper are older than 6000 B.C.E. (dates 26–28). Other sherds found in burials and middens with Kiffian decorative motifs and dates based on plant temper are younger than 6000 B.C.E. (dates 29, 32, 34, 45, 46, 52). Although these younger ages may be aberrant (EAAG), they are concordant with dates based on other materials when found in the same midden (dates 44–54; JFS). Likewise, two direct dates on bone harpoon points (dates 64, 65) are concordant with other dated material in refuse area 5 associated with the early Holocene occupational phase 2 (dates 64, 65). An additional pair of direct dates (dates 66, 68) on bone harpoon points found in situ in paleolake sediment, however, are more than 2 kyr younger and date to the middle of the mid-Holocene occupational phase 3. Additional testing of bone harpoon points is warranted to better understand these results.

## Results and Discussion

### Geologic Setting

Gobero is located on the northwestern rim of the Chad Basin, approximately 150 km southeast of the Aïr massif (Figure 1A). Isolated on a vast peneplain of mid-Cretaceous sandstone between fields of migrating barcan dunes (Figure 2), the most important cluster of sites at Gobero is located in low, calcrete-fringed paleodunes that are partially surrounded by paleolake deposits [Figure 1B, C). The paleodunes accumulated at Gobero over a period of at least seven millennia from the Late Pleistocene to the early Holocene (∼14,000–7000 B.C.E.), as determined by optically stimulated luminescence (OSL) dating of paleodune sand at various depths (Figure 1F, Table 1). During the best preserved intervals of occupation, the core paleodune sites (G1-3) formed islands that may have been originally partially conjoined as a narrow peninsula into Paleolake Gobero, a shallow, freshwater lake no more than 3 m in depth and 3 km in diameter (Figure 1C, D). The lake occupied a small endorheic basin located on a low rise between drainages southwest to the Niger River and southeast to Paleolake Chad. Fed periodically by surface water from the Aïr massif that pooled against a low east-west fault scarp (Mazelet) immediately to the south, Paleolake Gobero appears to have been more closely associated with the Chad Basin, as there is no trace of the giant catfish (*Arius gigas*) common to drainages of the Niger [21]. The archaeological sites were submerged when the lake filled to a depth greater than 5 m (Figure 1D). Over time the human bone in submerged burials darkened and hardened to resemble the pyrolusite (MnO_2_)-darkened vertebrate bone in the adjacent paleolake deposit.

### Model for the Gobero Sequence

**Figure 3:**
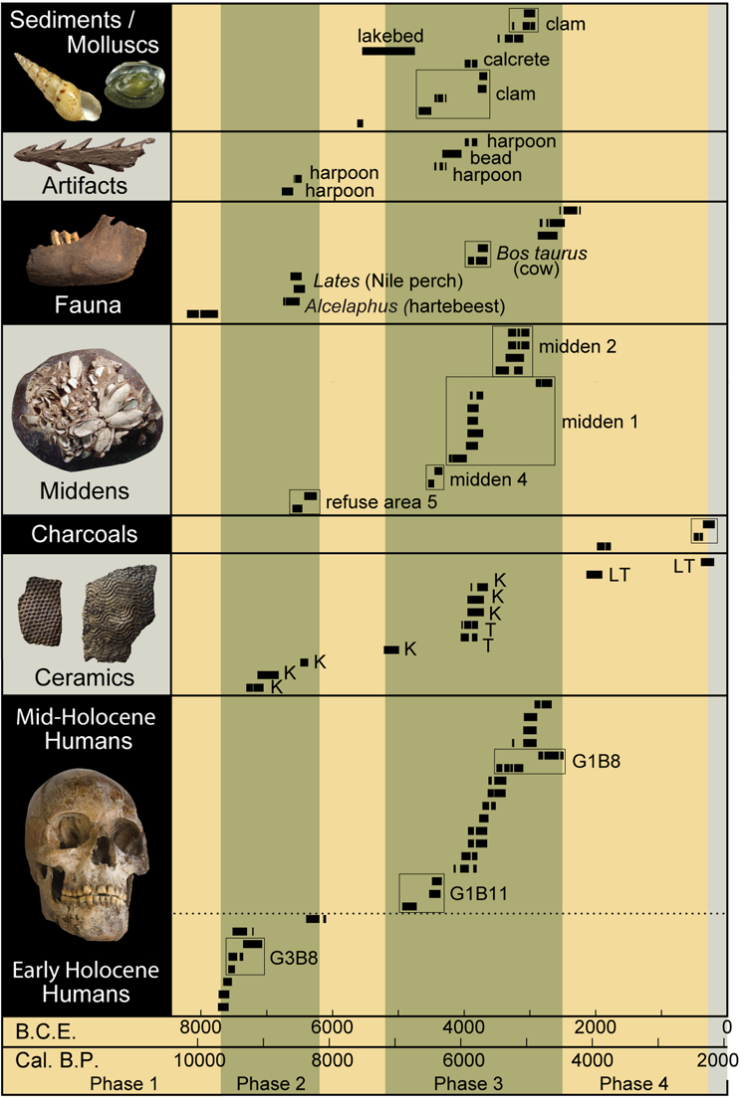
Radiocarbon (^14^C AMS) dates for human skeletons, ceramics, charcoals, middens, fauna, artifacts and sediment. Timelines and occupation phases 1–4 are shown at the bottom. Associated chronometric data are compiled in Table 2 using current atmospheric standards [55]. All of the burials that have been dated at Gobero fall within phases 2 and 3, which are shown as green to indicate favorable humid climate conditions; more arid intervals are shown as tan including occupation phases 1 and 4. Multiple dates on individual specimens or features are boxed. A dotted line separates early and mid-Holocene human burials. *Abbreviations*: *B.C.E.*, before current era (registered to calendar year zero); *B.P.*, before present (1950); *G1B8*, burial 8 on G1; *G1B11*, burial 11 on G1; *G3B8*, burial 8 on G3; *K*, Kiffian; *LT*, Late Tenerean; *T*, Tenerean.

Based on geochronological data (Tables 1, 2) with input from archaeological, craniometric, zooarchaeological and archaeobotanical analysis, we divide the record preserved at Gobero into four occupation phases (Figure 3).

#### Phase 1—Paleodune Accumulation (14,000–7700 B.C.E.)

Phase 1 is predominantly an arid interval at the close of the Pleistocene and beginning of the Holocene, when dune sands accumulated in the Gobero area over Cretaceous bedrock under climatic conditions characterized by weakened monsoons and the spread of aridification across northern Africa [4]–[6], [12]–[14]. The base of the paleodune deposit at site G1 dates to ∼14,000 B.C.E. (Figure 1B, F, section 12; Table 1, dates 3, 4). Although humid intervals are recorded elsewhere in the central Sahara [5], [13], [14] and may have occurred during phase 1 at Gobero, Paleolake Chad never grew to megalake dimensions until later in the early and mid-Holocene [5], [6], and the paleodune sequence at Gobero accumulated without a detectible hiatus. Deflated Ounanian artifacts [22]–[24] were recovered suggesting that during this phase there were transient hunter-gatherers in the Gobero area who seem not to have left any burial record in the lower portion of the paleodune sequence.

#### Phase 2—Early Holocene Occupation (7700–6200 B.C.E.)

**Figure 4:**
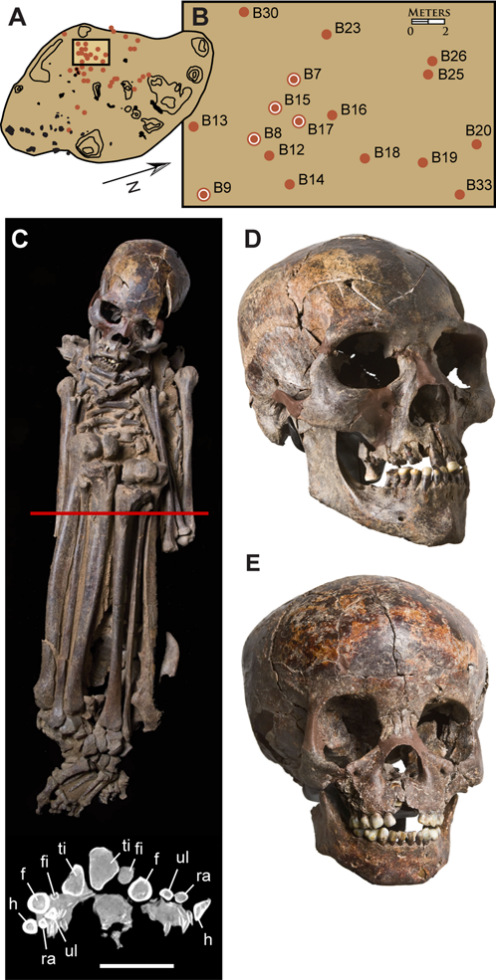
Early Holocene cemetery, burials and skulls. (A)-Gobero site G3 showing excavated burials (red dots). (B)-Enlarged map of the early Holocene cemetery showing the location of 17 undisturbed burials of skeletons with dark-stained bone (red dots). Five burials (red dot with outer ring) were directly dated to a narrow range of ∼7500±250 years B.C.E. (Figure 2; Table 2). (C)-Skeleton (dark-stained) of an early Holocene adult male (G3B8; ∼7515 B.C.E.) buried in supine, hyperflexed posture with hands over the mouth and feet crossed. Computed-tomography cross-section (below) across the middle of the skeleton (red line) shows the tightly bundled configuration of major limb bones (within a 25 cm×12 cm rectangle) for an adult with stature approximately 2 m. (D)-Skull of early Holocene adult male (as in C) showing long, low calvarium, broad zygomatic width and relatively flat face. (E)-Skull of an early Holocene juvenile (G3B17b; ∼7630 B.C.E; estimated age 5 years) already showing long, low cranial proportions. Scale bar in C equals 13.3 cm for skeleton and 10 cm for CT scan; skull length (glabella-opisthocranion) in D and E equals 190.0 mm and 171.0 mm, respectively. *Abbreviations*: *f*, femur, *fi*, fibula; *h*, humerus; *r*, radius; *ti*, tibia; *ul*, ulna.

During phase 2, wet climatic conditions attracted a population of hunter-fisher-gatherers to Gobero sites, which served both funerary and habitation functions. In one area of site G3 no larger than 50 m^2^, 17 closely interspaced (≤4 m), undisturbed burials contain dark-stained skeletons composing a cemetery (Figure 4A, B). Direct dates for five of these burials indicate an age of ∼7500 B.C.E. and range over only ∼250 years (Figure 3; Table 2, dates 1–7). This cemetery is considerably larger than a small cluster of burials at a somewhat younger site along the upper Nile (El Damer) [11] and predates by three millennia the oldest cemetery in Egypt's Western Desert (Gebel Ramlah) [25]. Phase 2 is delimited in time by burials within site G3 with direct dates (Table 2, dates 1, 7), the oldest a subadult individual in the cemetery (7730–7580, midpoint 7655 B.C.E.) and the youngest an adult located nearby (6380–6210, midpoint 6295 B.C.E.).

**Figure 5:**
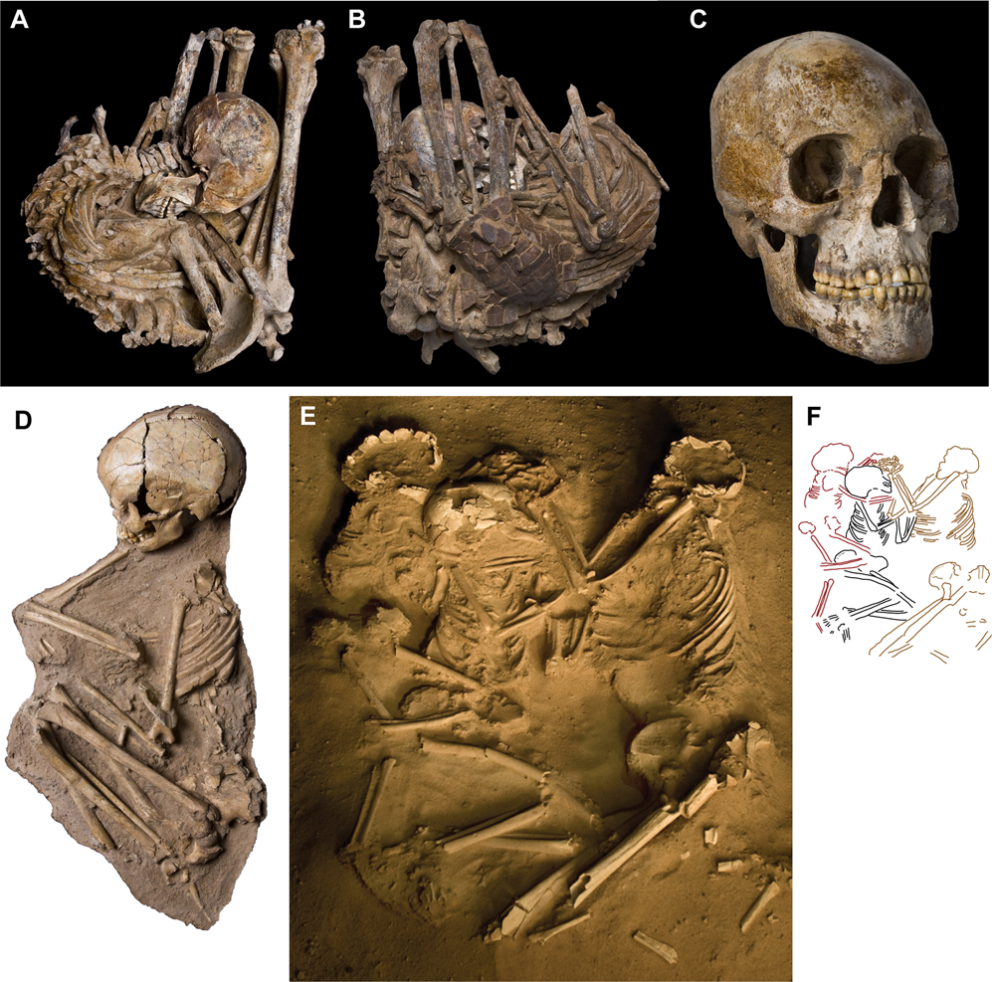
Mid-Holocene burials and skull. (A)-Top view of mid-Holocene adult male (G1B11; ∼4645 B.C.E.) buried in a recumbent hyperflexed posture. (B)-Bottom view of burial in A showing a mud turtle carapace (*Pelusios adansonii*) in contact with the ventral aspect of the pelvic girdle. (C)-Skull from burial in A and B showing high calvarium, narrow zygomatic width and more prognathous face. (D)-Mid-Holocene juvenile (G1B2; ∼2835 B.C.E.) with upper arm bracelet of hippo ivory. (E)-Mid-Holocene triple burial involving an adult female (G1B8; ∼3315 B.C.E.) and two juveniles (G1B9, G1B10) with intertwined arms, hands and legs. (F)-Schematic showing skeletal positions in the triple burial with the adult female on right (tan, G1B8) facing juveniles with estimated ages of 8 years (black, G1B9) and 5 years (red, G1B8).

Phase 2 peoples are tall in stature, approaching two meters for both males and females. Hyperflexed, supine burial postures predominate, their compact configuration and anatomical articulation suggesting that their bodies were tightly bound with animal skin, ligament or basketry binding, although no trace of these perishable materials are preserved (Figure 4C). Their crania are long and low and are characterized by a distinct occipital bun, flattened sagittal profile, pentagonal posterior outline, broad proportions across the zygoma and interorbital region, broad nasal aperture, and negligible alveolar prognathism (Figure 4D), features that are apparent in juveniles as young as four years of age (Figure 4E) and absent in skulls from mid-Holocene burials (Figure 5C).

**Figure 6:**
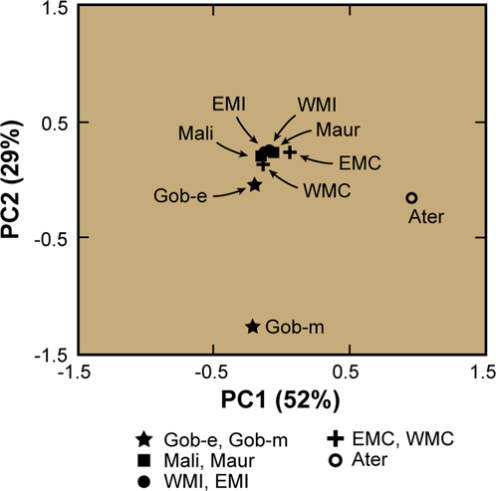
Principal components analysis of craniofacial dimensions among Late Pleistocene to mid-Holocene populations from the Maghreb and southern Sahara. Plot of first two principal components extracted from a mean matrix for 17 craniometric variables (Tables 4, 7) in 9 human populations (Table 3) from the Late Pleistocene through the mid-Holocene from the Maghreb and southern Sahara. Seven trans-Saharan populations cluster together, whereas Late Pleistocene Aterians (Ater) and the mid-Holocene population at Gobero (Gob-m) are striking outliers. Axes are scaled by the square root of the corresponding eigenvalue for the principal component. *Abbreviations*: *Ater*, Aterian; *EMC*, eastern Maghreb Capsian; *EMI*, eastern Maghreb Iberomaurusian; *Gob-e*, Gobero early Holocene; *Gob-m*, Gobero mid-Holocene; *Mali*, Hassi-el-Abiod, Mali; *Maur*, Mauritania; *WMC*, western Maghreb Capsian; *WMI*, western Maghreb Iberomaurusian.

Craniometric data from seven human groups (Tables 3, 4) were subjected to principal components analysis, which allies the early Holocene population at Gobero (Gob-e) with mid-Holocene “Mechtoids” from Mali and Mauritania [18], [26], [27] and with Late Pleistocene Iberomaurusians and early Holocene Capsians from across the Maghreb (see cluster in Figure 6). The striking similarity between these seven human populations confirms previous suggestions regarding their affinity [18] and is particularly significant given their temporal range (Late Pleistocene to mid-Holocene) and trans-Saharan geographic distribution (across the Maghreb to the southern Sahara).

**Table 3:** Nine human populations sampled for craniometric analysis ranging in age from the Late Pleistocene (ca. 80,000 BP, Aterian) to the mid-Holocene (ca. 4000 BP) and in geographic distribution across the Maghreb to the southern Sahara [18], [19], [26], [27], [54].

No.	Sample Size	Area, Form/Culture (Age)	Acronym or Abbreviation	Sites
**1**	6	Sahara, Aterian (Late Pleistocene)	Ater	TémaraDar-es-Soltan
**2**	39	Eastern Maghreb, Iberomaurusian (Late Pleistocene)	EMI	Kef-oum-TouizaAli-BachaAfalou-bou-Rhummel
**3**	5	Western Maghreb, Iberomaurusian (Late Pleistocene)	WMI	TaforaltTaza IRachgoun 4La Mouillah
**4**	14	Eastern Maghreb, Capsian (early Holocene)	EMC	Ain DokkaraKhanguet-el-MouhaadMedjez IIAin MeterchemAioun BericheGrotte des HyenesMechta-el-ArbiGambetta
**5**	22	Western Maghreb, Capsian (early to mid-Holocene)	WMC	Columnata
**6**	69	Mauritania, “Mechtoid” (mid-Holocene)	Maur	IzritenSebkha LaasailiaSebkha AmtalSebkha MahariatSebkha LemheirisSebkha EdjailaTintanChami
**7**	45	Mali, “Mechtoid” (mid-Holocene)	Mali	Hassi-el-AbiodAsselar
**8**	6	Gobero, Kiffian (early Holocene)	Gob-e	Gobero (G3)
**9**	12	Gobero, Tenerean (mid-Holocene)	Gob-m	Gobero (G1-3, G5)

**Table 4:** Craniometric means for human samples.

Measure-ment Acronym	Ater	WMI	WMC	EMI	EMC	Maur	Mali	Gob-e	Gob-m
**LGO**	197.88	189.17	185.50	187.51	189.04	189.11	187.19	188.00	181.64
**BPX**	149.38	143.18	143.45	143.59	144.56	143.48	143.29	141.67	131.83
**LBN**	112.00	107.00	101.50	106.81	106.86	106.57	105.87	102.33	103.71
**HBB**	141.50	135.83	135.00	135.72	137.98	136.15	135.41	134.00	137.67
**AFR**	139.50	126.10	128.70	126.03	129.28	126.89	125.59	128.00	126.11
**APA**	130.67	133.50	128.09	132.23	131.60	132.91	131.75	133.60	131.50
**AOC**	122.33	123.59	122.56	123.46	123.11	123.41	123.39	121.67	110.43
**CFR**	119.00	111.02	113.11	110.30	112.89	111.51	110.19	111.25	111.56
**COC**	103.00	103.24	102.00	102.86	102.87	103.08	102.69	99.67	96.71
**HNP**	72.00	64.89	69.75	63.97	66.25	65.60	64.31	68.00	62.83
**HNZ**	49.25	51.41	51.75	49.68	49.92	51.19	49.84	53.50	49.64
**BNZ**	27.00	27.38	24.80	26.73	26.21	27.02	26.69	28.60	27.85
**BZY**	152.00	139.43	143.00	135.71	138.43	140.10	136.42	141.67	145.80
**BFW**	107.95	93.65	92.27	94.56	96.51	94.17	94.41	94.50	95.07
**BFX**	118.33	120.05	115.82	118.35	118.43	119.50	118.10	121.50	109.11
**HORC**	550.00	537.88	523.38	534.56	536.74	536.84	532.84	532.00	485.80

Craniometric means for early and mid-Holocene skulls from Gobero and comparative samples elsewhere from northern Africa. *Measurement acronyms* (after [18]): *LGO*, glabella-occipital length; *BPX*, maximum cranial breadth; *LBN*, basion-nasion; *HBB*, basion-bregma; *AFR*, frontal arc; *APA*, parietal arc; *AOC*, occipital arc; *CFR*, frontal chord; *COC*, occipital chord; *HNP*, nasion-prosthion; *HNZ*, nasion-nasospinale; *BNZ*, nasal breadth; *BZY*, bizygomtic breadth; *BFW*, minimum frontal breadth; *BFX*, maximum frontal breadth; *HORC*, horizontal circumference above the superciliary arches. *Sample acronyms and abbreviations*: *Ater*, Aterian; *EMI*, eastern Maghreb Iberomaurusian; *EMC*, eastern Maghreb Capsian; *Gob-e*, Gobero early Holocene; *Gob-m*, Gobero mid-Holocene; *Maur*, Mauritania; *Mali*, Hassi-el-Abiod, Mali; *WMI*, western Maghreb Iberomaurusian; *WMC*, western Maghreb Capsian.

**Figure 7:**
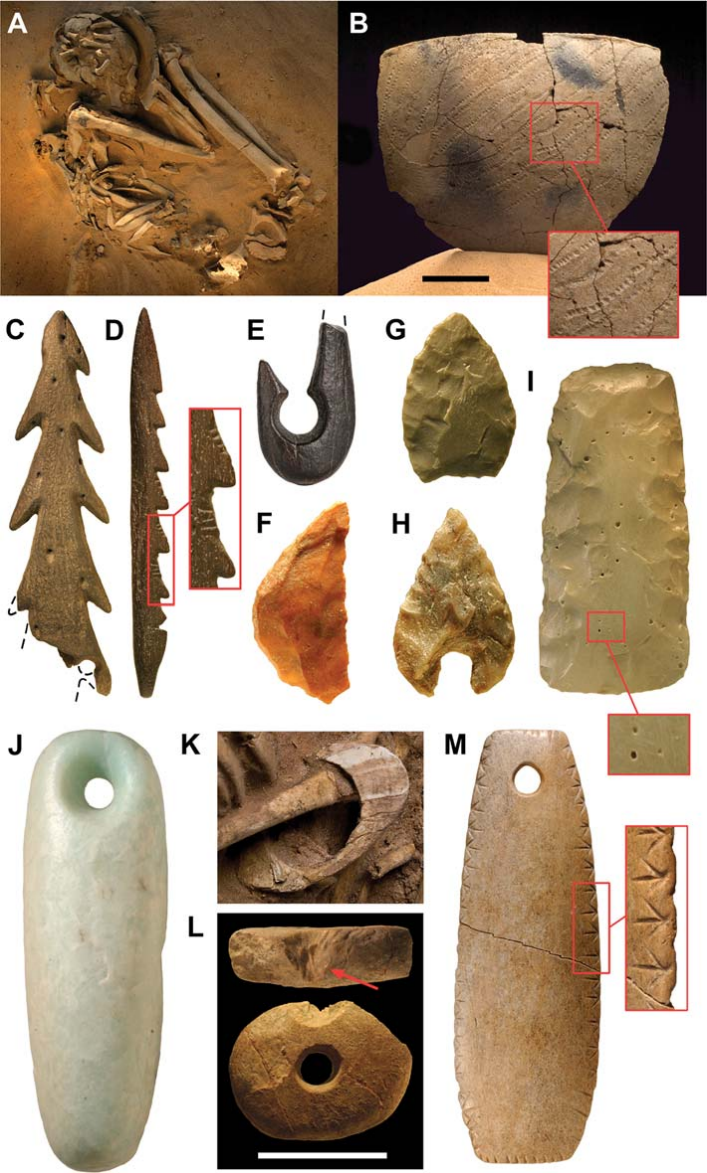
Ceramic, lithic, bone and hippo ivory artifacts and ornaments. (A)-Mid-Holocene adult male (G3B36; ∼3500 B.C.E.) buried with skull resting in a partial ceramic vessel (see B). (B)-Side and magnified view of ceramic vessel (G3-94) under skull (see A) showing rocker stamp decoration. Kiffian tool kit (C–F). (C)-Biserial bone harpoon point with perforated butt (GA154) made from a crocodile dentary. (D)-Uniserial fixed barbed point with notched butt (GA130) made from an artiodactyl long bone. (E)-Bone hook (GA31a). (F)-Crescent-shaped microlith (G1-71b) from site G1 (deflated). Tenerean tool kit (G-I). (G)-Felsite bifacial point (G3-1b) associated with an adult male burial (G3B4). (H)-One (G1-134) of four hollow-based points associated with a mid-Holocene adult female (G1B8; ∼3315 B.C.E.) in a triple burial (Figure 3E, F). (I)-Anterior and magnified view of a felsite adze (GA110c) showing the green color and vesicles common to this source rock. (J)-Amazonite pendant (GA124). (K)-Upper arm bracelet (G1-7) carved in hippo ivory near the distal end of the left humerus in a juvenile burial (G1B2; ∼2835 B.C.E.). (L)-Bead (G3-6 necklace, bead 9) made of hippo ivory showing the paired bite mark from the incisors of a rodent (top, arrow) on a divot removed from the bead margin (bottom). (M)-Anterior and magnified lateral views of a pendant (part of G3-6 necklace) carved in hippo ivory and found in situ on a mid-Holocene adult female (G3B41; ∼3620 B.C.E.). Scale bars equal 5 cm in B and 2 cm in L. Ages given above are from ^14^C AMS dates on enamel bioapatite and represent the midpoint of the calibrated radiocarbon confidence interval (Table 2). Maximum artifact length is 11.9 cm in C, 13.2 cm in D, 2.0 cm in E, 2.3 cm in F, 2.04 cm in G, 2.0 cm in H, 8.2 cm in I, 4.4 cm in J, 8.4 cm in K, and 8.8 cm in M.

Microliths, bone harpoon points and hooks, and ceramics with dotted wavy-line and zigzag impressed motifs were found in the burial fill, in an associated refuse area, and in nearby paleolake deposits (Figure 7C–F). These artifacts exhibit attributes of the Kiffian technocomplex, named after the type site Adrar-n-Kiffi at Adrar Bous some 500 km to the north [28], [29]. Direct dates on bone harpoon points and plant temper in Kiffian sherds confirm the association of several of these artifacts with this occupational phase (Figure 3; Table 2, dates 26–28, 64, 65). Nile perch (*Lates niloticus*) and large catfish dominate the midden fauna, which also includes bones and teeth from hippos, several bovids, small carnivores, softshell turtles and crocodiles (Table 5, refuse area 5). The burial density, tool kit, ceramics, and midden fauna suggest a largely sedentary population with a subsistence economy based on fishing and on hunting of a range of savanna vertebrates.

**Table 5:** Faunal list at Gobero showing taxa recorded at archaeological sites (G1-3), in early and mid-Holocene middens (middens 1–4, refuse area 5), and in the Gobero area (in paleolake deposits or on deflation surfaces).

Higher Taxon	Tribe, Genus, Species	Sites G1-3	Mid-Holocene Middens 1–4	Early Holocene Refuse Area 5	Gobero Area
**MOLLUSCA**					
Gastropoda	*Melanoides tuberculata*	+	**x**		+
Pelecypoda	*Mutela* sp	**x**	**x**	+	+
	?*Aspatharia* sp	+	+	+	+
**ACTINOPTERYGIA**					
Polypteridae	*Polypterus* sp.		+	+	
Mormyridae			+	+	
	*Gymnarchus niloticus*		+		
Cyprinidae			+	+	
Osteoglossidae	*Heterotis niloticus*		+	+	
Siluriformes		**x**	+	**x**	**x**
Bagridae			**x**	+	+
	*Auchenoglanis* sp.			+	
Claridae	*Clarias* sp.	+	**x**	**x**	+
Mochocidae	*Synodontis* sp.		+		+
Centropomidae	*Lates niloticus*	+	**x**	**x**	**x**
Cichlidae	Tilapinii	**x**	**x**	**x**	+
**AMPHIBIA**					
Bufonidae	Gen. et sp. indet.	+			
**REPTILIA**					
Testudines		+		+	
Pelomedusidae	*Pelusios adansonii*	+	**x**	+	+
Trionychidae		+	+	**x**	+
	*Cyclanorbis senegalensis*				+
	*Trionyx triunguis*				+
Varanidae	*Varanus* sp.		+		+
Pythonidae	*Python sebae*	+	+		+
Crocodylidae	*Crocodylus niloticus*	+	+	**x**	+
**AVES**					
Palaeognathae	*Struthio camelus*	+^1^	+^1^		
Neognathae		+		+	+
**MAMMALIA**					
Rodentia		+	+	+	+
	*Thryonomys swinderianus*	+		+	
Felidae	*Felis sylvestris*			+	
	*Panthera leo*				+
Herpestidae				+	
Lutrinae					+
Canidae	*Canis aureus*		+		
	*Lycaon pictus*				+
	*Hyaena hyaena*				+
Elephantidae	*Loxodonta africana*				+
Suidae	*Phacochoerus aethiopicus*	+			+
Hippopotamidae	*Hippopotamus amphibius*	**x**	+	+	+
Giraffidae	*Giraffa camelopardalis*				+
Bovidae	*Gazella* sp.			+	+
	*Gazella rufifrons*				+
	*Gazella dama*				+
	*Tragelaphus scriptus*	+			+
	*Tragelaphus spekei*				+
	*Tragelaphus strepsiceros*				+
	*Taurotragus derbianus*				+
	*Redunca redunca*	+	+	+	+
	*Kobus ellipsiprymnus*	+			+
	*Kobus kob*			+	+
	*Hippotragus equinus*				+
	*Oryx dammah*				+
	*Alcelaphus buselaphus*				+
	*Syncerus caffer*	+			+
	*Bos taurus* (domesticate)		+		+

*Abbreviations*: +, species present; **x**, species very common.

1 As eggshell beads.

Pollen from phase 2 burials at site G3 indicates an open, low-diversity savanna with grasses and sedges, and an arboreal component including fig (*Ficus*) and tamarisk (*Tamarix*). Hydrophytes and rushes (*Juncus*) suggest the presence of permanent water and marshy habitats [15], [30]. Nevertheless, xerophytes including saltbushes (Chenopodiaceaea) are significant, indicating that sandy habitats were also present, although perhaps distant, in the region.

Toward the end of phase 2 (∼6500–6300 B.C.E.), the level of Paleolake Gobero rose, at least episodically, submerging the paleodunes and forcing the relocation of occupants (Figure 1D). Well-aerated permanent water at depths of 5 m or more are suggested by large vertebrae (up to 5 cm diameter) of the Nile perch (*Lates niloticus*), which correspond to a body length of up to 2 m [31]. These vertebrae were found in situ in paleolake sediments and directly dated to the end of this phase (Figure 3; Table 2, dates 57, 58; midpoint average 6525 B.C.E.). The darkened bone color of all human skeletons in phase 2 burials is indicative of sustained inundation. Inundation, nevertheless, may have been episodic, as a dark-stained human skeleton (G3B28), Kiffian potsherd, refuse area, and hartebeest skeleton (*Alcelaphus buselaphus*) are also directly dated to this interval (Figure 3; Table 2, dates 8, 28, 40, 41, 56; midpoint range 6540–6295 B.C.E.).

Because spillways external to the restricted catchment for Paleolake Gobero may have kept inundation levels under 10 m (Figure 1D), it is possible that floodwaters may not have displaced lakeside occupants by a long distance or over a long temporal span. The inundation history at Gobero is doubtless complex, as the earliest mid-Holocene burials are also variegated or darkly stained (Table 2, dates 9–13; midpoint range 4825–3860 B.C.E.). Resolving additional inundation events of short duration will require further direct dates on paleolake fauna and sediments.

#### Occupational Interruption (6200–5200 B.C.E)

A harsh arid interval separates early and mid-Holocene populations at Gobero, when the paleolake appears to have dried out and the area abandoned. Although we have no means to directly assess aridity, no terrestrial or aquatic vertebrates or lakebed sediment have been dated to this interval, which lasted approximately one millennium (Figure 3). The only specimens dated within this interval were found in the paleolake deposit and consist of a cluster of the small gastropod *Melanoides tuberculata*, a species that prefers periodically flooded habitats to permanent water bodies.

This occupational hiatus correlates well with the “arid interruption” in the central Sahara [12], a somewhat shorter interval (∼6400–6000 B.C.E.; ∼400 yr) of severe climatic deterioration across the Chad Basin [5], [12] linked to cooling events in the North Atlantic [13], [14]. This overlaps the early portion of the occupational hiatus at Gobero, the beginning of which is set after the youngest burial dated so far in occupation phase 2 (Figure 3; Table 2, date 8, 6380–6210 B.C.E., midpoint 6295 B.C.E.). The end of the occupational interruption is set just before direct dates that indicate the return of humid conditions, including paleolake sediment 20–25 cm below the fossil-rich zone (Figure 1B, E, section 9; Figure 3; Table 2, date 75, 5550–4750 B.C.E., midpoint 5150 B.C.E.), a ceramic sherd from burial fill in site G1 (Figure 3; Table 2, date 29, 5220–5000 B.C.E., midpoint 5110 B.C.E.), and the oldest burial dated so far from occupation phase 3 (Figures 3, 5A–C; Table 2, enamel dates 9, 10, midpoint average 4635 B.C.E.).

#### Phase 3—Mid-Holocene Occupation (5200–2500 B.C.E.)

Phase 3 is a long interval over two millennia in length that witnessed the return of humid conditions and the re-occupation of Gobero by humans that account for approximately one-half of excavated burials (Figure 3; Table 2, dates 9–25). The lower boundary of this phase marks the end of the occupational interruption and, as discussed above, is based on dates on lakebed sediment, a potsherd from burial fill, and the oldest burial in the occupation phase (Figures 3, 5A–C). The oldest skeletons within this phase have variegated or dark-stained bone and appear to have undergone episodes of inundation postdating their internment (Figure 5A–C, Table 2, dates 9–13). Occupational phase 3 at Gobero comes to a close with the youngest dated burial, a subadult approximately 11 years old (Figures 3, 5D; Table 2, date 25, 2910–2760 B.C.E., midpoint 2835 B.C.E.).

Phase 3 humans have more gracile skeletons and shorter stature for both males and females. They are buried most commonly in semi-flexed postures on either left or right sides (Figure 5D, E). Their crania are long, high and narrow, and their faces are taller with considerable alveolar prognathism (Figure 5C). Principal components analysis of craniometric data clearly distinguishes the mid-Holocene population at Gobero (Gob-m) from all other sampled populations, including the early Holocene population at Gobero, Iberomaurusian and Capsian populations from the Maghreb, “Mechtoids” from Mali and Mauritania, as well as much older Aterian samples (Figure 6). The morphological isolation of the mid-Holocene population from Gobero is particularly noteworthy, as several of the other populations sampled (WMC, Mali, Maur) are believed to be mid-Holocene contemporaries.

Grave goods occur in approximately 20% of the burials excavated thus far (7 of 35 burials) and include bones or tusks from wild fauna, ceramics, lithic projectile points, and bone, ivory and shell ornaments (Figures 5B, 7A, B, H, K–M). Although the disc knife that characterizes the mid-Holocene Tenerean industry [28], [29] has never been recovered in situ, small projectile points that also characterize the Tenerean tool kit are present in some burials that are directly dated to the mid-Holocene (Figure 7H; Table 2, date 20, 3500–3130 B.C.E., midpoint 3315 B.C.E.). Burials of particular note include an adult male with his skull resting on a half vessel decorated with an alternately pivoting stamped impression (Figure 7A, B; Table 2, date 18, midpoint 3500 B.C.E). Another adult male was buried in a recumbent pose seated on the carapace of a mud turtle (Figure 5A–C; Table 2, dates 9, 10, midpoint average 4635 B.C.E.). A triple burial, composed of an adult female and two juveniles, has intertwined arms, hands and legs, suggesting that they died nearly simultaneously and were interred in an intimate pose within a short period of time (Figure 5E, F; Table 2, date 20, midpoint 3315 B.C.E). Four hollow-based points lie between their limbs and underneath their skeletons (Figure 7H), and pollen clusters from flower heads of the wool flower (*Celosia*) were detected in underlying sediment. Like other burials at Gobero, these individuals show no sign of a violent traumatic death.

A conspicuous fine-grained green rock (with tan and brown variants) was often used for points, scrapers and adzes (Figure 7G, I) and always used for the delicate Tenerean disc knives [28], [29] at sites east and south of the Aïr massif. Previously this distinctive rock was identified as microcrystalline quartz, initially as jasper [32] and later as silicified vitric tuff [28]. For some 50 years, its source has remained a matter of speculation. Along with amazonite, the green rock was cited as evidence of trade over distances of one thousand kilometers or more, in order to link the Aïr with Tibesti (northern Chad-southern Libya) or regions farther afield [28], [33], [34].

**Figure 8:**
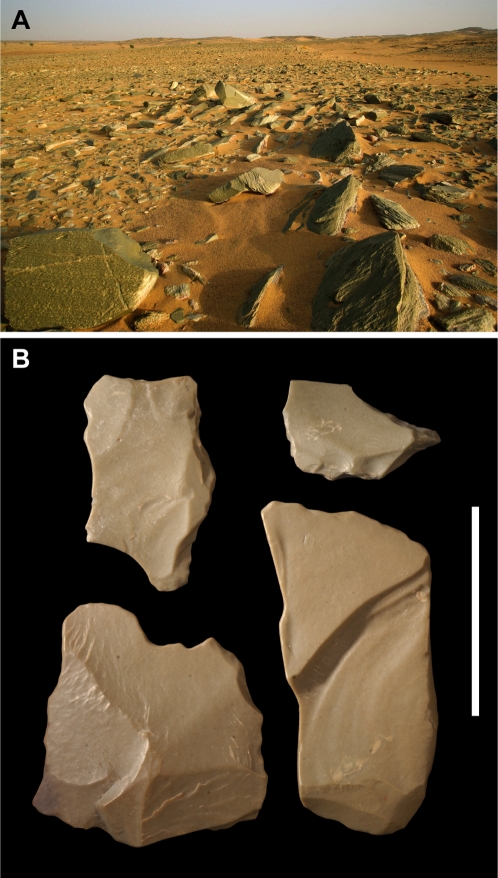
Alallaka, a felsite knapping site on the edge of the Aÿr massif. (A)-An intrusion of green microcrystalline feldpar, or felsite [35], is exposed as an oval outcrop approximately 0.8 km in width and 2.5 km in length near the Alallaka wadi on the southeastern edge of the Air massif (an area known as Takolokouzet), situated 160 km north of the Gobero site complex Figure 1A). The exposed rock shows the characteristic green hue, variable lamination, and vesicular texture common to many of the felsite lithics from Gobero. (B)-Abundant debitage as well as large groundstones attest to a longstanding knapping operation at Alallaka. Scale bar equals 3 cm.

Thin sections of artifacts made from the green rock and its color variants show the near absence of quartz. It is correctly identified as a felsite, a fine-grained volcanic rock composed of microcrystalline feldspar [35]. During reconnaissance a short distance (160 km) north of Gobero on the edge of the Aïr massif, we located a narrow outcrop of green felsite rock near the Alallaka wadi (Figures 1A, 8A). The site is littered with debitage from a longstanding knapping operation (Figure 8B). The frequent use of this rock at Gobero and Adrar Bous and its availability in nearby wadis along the eastern edge of the massif (Takolokouzet highland) suggests there were multiple felsite sources local to the Aïr. Given the absence of thorough geologic reconnaissance in the Aïr or any comparative trace element analysis, neither felsite nor amazonite provides evidence to infer trans-Saharan trade in the early or mid-Holocene.

At least semi-sedentary occupation is inferred from the number and density of undisturbed mid-Holocene burials, lack of evidence from strontium isotope analysis for enhanced mobility, abundance of lithic debitage on site, presence of groundstones, and numerous rodent marks on human bone and ornaments indicative of commensal species (Figure 7L) [36], [37]. The prevalence of juveniles of very young age among the interred also favors longer term occupation by family groups over transient visitation of the area as a watering hole or attractive hunting ground.

**Figure 9:**
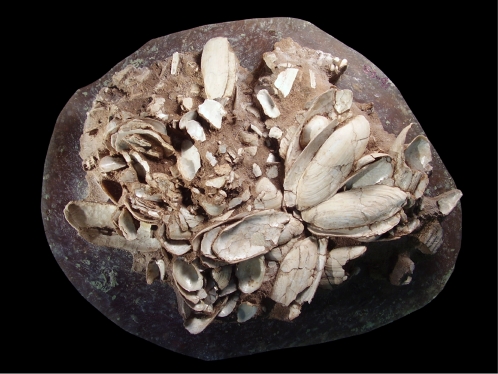
Mid-Holocene midden. Portion of a mid-Holocene midden (midden 4) with matrix removed showing stacking of the valves of the clam Mutela, articulated fish vertebrae, and potsherds (Table 2, dates 42, 43, average midpoint ∼4445 B.C.E.).

Clams (*Mutela*), small catfish (*Clarias*) and tilapia dominate the midden fauna (Figure 9), which also includes bones and teeth from hippos, a small antelope, small carnivores, softshell turtles and crocodiles (Table 5; middens 1–4). Domesticated cattle (*Bos taurus*) are present but unlike Adrar Bous [28], [29] comprise only a minor component of the midden and area fauna (Figure 3, *Bos* partial mandible on chart sidebar; GF10). The scarcity of bones or teeth of domesticated cattle suggests a subsistence economy emphasizing fishing in shallow waters and hunting of a range of savanna vertebrates. The gathering of grain and cattle pastoralism may also have played important nutritional or economic roles; further data relevant to occupancy and subsistence patterns are needed, such as seasonality data from piscine otoliths recovered from middens. Elsewhere in the southern Sahara, diversification of dietary resources that combine gathering, hunting, fishing and pastoralism seems to occur under less certain climatic conditions [34], [38], [39], a pattern that may well accommodate the emerging archaeological record during the mid-Holocene at Gobero.

**Figure 10:**
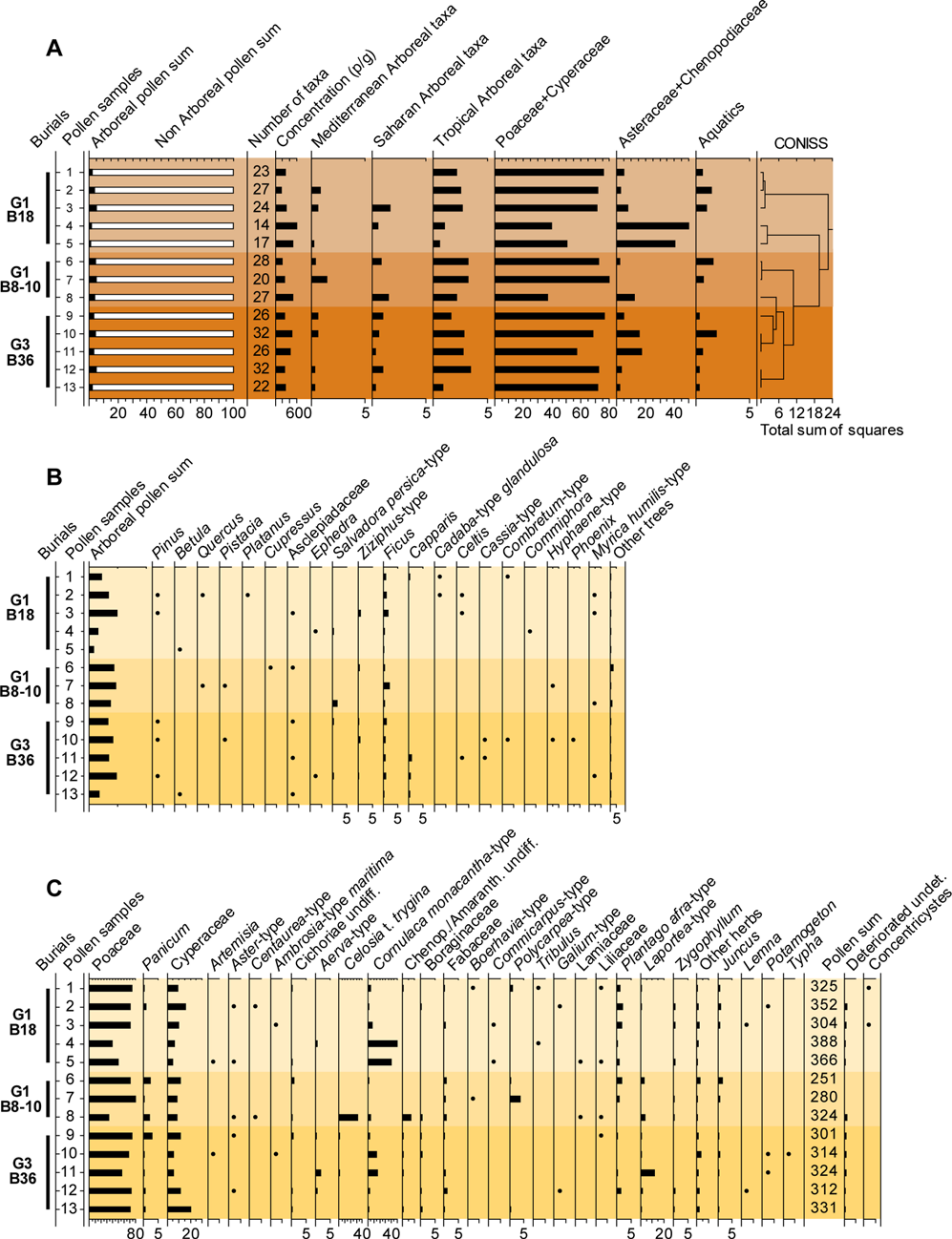
Pollen composition in mid-Holocene burials. (A)-Percentage pollen spectra from three mid-Holocene burials on sites G1 and G3 showing low diversity vegetation and the prevalence of Sahelian phytogeographical character. (B)-Arboreal pollen. (C)-Non-arboreal pollen. Long distance transport is low, as indicated by the paucity of Mediterranean taxa [15]. Crushed or damaged pollen grains that could be identified were summed in the relevant taxa. Only pollen that could not be identified were included in the deteriorated sum. *Abbreviations*: *CONISS*, constrained incremental sum of squares; *p/g*, pollen per gram.

Pollen spectra from phase 3 burials indicate a mosaic of habitats. Open savannas with shrubland and grassland vegetation dominated, with sporadic presence of a fairly diversified Sudanian and tropical tree flora (Figure 10). Plants linked to wet environments include hydrophytes, which indicate the presence of shallow freshwater lakes. Xeric and psammophilous plants indicate the presence of sandy soils.

#### Phase 4—Transient Presence (2500–300 B.C.E)

Phase 4 marks the onset of widespread aridification in the Sahara [4], [5], [7], [8], [12]–[14] and brings to a close the record at Gobero. Several undecorated pots, one with an underlying hearth, have been directly dated to this interval (Figure 3; Table 2, dates 35, 36, 38, 39), suggesting the transient presence of more nomadic cattle herders. As with phase 1, there are no burials at Gobero dating to phase 4.

#### Conclusions

In a recent (2003) review of Niger's archaeological record [40], the near absence of human remains across the broad expanse of the hyperarid Ténéré Desert was noted. Like many other regions of the Sahara, humid intervals in the early and mid-Holocene created attractive areas for human settlement in Niger. The Gobero site complex, which includes as many as 200 burials, underscores the scale and complexity of human occupation in a “greener” Sahara as well as the fragility of that record under present conditions. We draw the following major inferences from the rich and relatively continuous Holocene record at Gobero:

1. *Early Holocene sedentism*. The early Holocene occupants at Gobero (7700–6300 B.C.E.) were largely sedentary hunter-fisher-gatherers with lakeside funerary sites that include the earliest recorded cemetery in the Sahara dating to ∼7500 B.C.E.
2. *Trans-Saharan craniometry*. Principal components analysis of craniometric variables closely allies the early Holocene occupants at Gobero, who were buried with Kiffian material culture, with Late Pleistocene to mid-Holocene humans from the Maghreb and southern Sahara referred to as Iberomaurusians, Capsians and “Mechtoids.” Outliers to this cluster of populations include an older Aterian sample and the mid-Holocene occupants at Gobero associated with Tenerean material culture.
3. *Arid interruption*. Early and mid-Holocene occupation phases 2 and 3 at Gobero are separated in time by a barren interval (6200–5200 B.C.E), which is associated with a period of severe aridification recorded across the Sahara.
4. *Dietary diversification*. Diversification of dietary resources, perhaps in response to increasing or episodic aridification, characterizes mid-Holocene subsistence strategies at Gobero (5200–2500 B.C.E.), as reflected in dated middens containing clams, fish, wild bovids and domesticated cattle.
5. *Population replacement*. Population replacement rather than gradual phenotypic evolution best explains the distinctive craniofacial morphology and funerary practices of the human occupants during phases 2 and 3 in the early and mid-Holocene, respectively, particularly considering the relatively short intervening occupational hiatus.
6. *Regional differentiation*. The timing of population change observed at Gobero may only characterize a restricted area. Other areas in the southern Sahara, even those with comparable environmental conditions such as Hassi-el-Abiod in Mali, appear to show a later transition between human populations. The data from Gobero, when combined with existing sites in North Africa, indicate we are just beginning to understand the complex history of biosocial evolution in the face of severe climate fluctuation in the Sahara, a vast region that was occupied for much of the Holocene by an anatomically diverse series of human populations.

## Materials and Methods

### Discovery and Excavation

The Gobero site complex was discovered in 2000 during paleontological reconnaissance in central Niger, briefly revisited in 2003, and noted in the press the following year [41]. The *Gobero Archaeological Project* was established to coordinate field work and research by an international, multidisciplinary team. Two field seasons (2005, 2006) have been undertaken, during which 67 burials were excavated and the main paleodune burial sites and adjacent paleolake deposits were mapped and sampled. A minimum of 182 human burials are preserved in the central area of the Gobero site complex (G1-5) buried within paleodunes on the edge of a paleolake. With few exceptions, only those burials that have reached the surface have been mapped and/or excavated thus far, and the total number of burials will very likely exceed 200 when the site complex is fully explored and excavated. The paleodunes and lakebed sites also preserve several middens, fossils of high quality documenting the invertebrate and vertebrate faunal record, and a pollen record.

After setting a corner datum stake, a total station was used to map each site using a square meter grid. The total station was also used to integrate geologic sections and to log several topographic profiles across the paleolake catchment. Given the absence of any obvious bedding planes in the paleodune sediment or grave pit boundaries around any of the burials, excavation proceeded by dividing the sediment into a surface layer and burial levels of set depth. All sediment removed was screened (3 and 4 mm sieves), and lithics, ceramics and skeletal fragments were collected and labeled according to burial, level and grid square. The total station was also used to log positional data for several skeletal landmarks as well as significant grave goods. Sediment samples were taken for biogeochemical, pollen and parasite analysis.

Burials of significance for interpreting funerary practices were removed in plaster jackets. Individuals were excavated according to the general site protocol (outlined above) until the integrity of the specimen was at risk. All pollen and parasite sampling was then completed and the skeleton was collected intact using conservation grade consolidant (B72) and an encasing jacket of plaster. Laboratory preparation of these specimens was conducted under a stereomicroscope in the Fossil Lab at the University of Chicago, resulting in detailed preservation of skeletal position and the relationship between the skeleton and particular grave inclusions. Burials collected using this protocol include an adult male in hyperflexed supine pose (Figure 4C), an adult male buried in recumbent pose and associated with the carapace of a mud turtle (Figure 5A–C), a triple burial of an adult female and two juveniles (Figure 5E, F), a juvenile in vertical burial pose, and a juvenile with an upper arm bracelet (Figure 5D).

### Optically Stimulated Luminescence Dating of Paleodune Sand

When grains of quartz are hidden from exposure to light, they accumulate trapped charges due to ionizing radiation from radionucleotides in the sediment. This energy accumulates predictably over time and is released as luminescence after controlled light stimulation in the lab. The age of the sediment (last exposure to daylight) is a measure of the paleodose (radiation dose to which the crystalline material has been exposed) divided by the total radiation dose for each year [42], [43]. For the paleodune samples from Gobero, the cosmic radiation dose was estimated assuming a sediment water content of 5±2% at an altitude of 560 m. Samples were collected on a moonless night from a newly excavated section of paleodune sediment, with a depth ranging from 1–3 m below the paleodune surface (Table 1).

### Direct ^14^C AMS Dating of Enamel and Bone

Bone collagen is usually thoroughly degraded in arid environments, which is true for all skeletal samples from Gobero (human, faunal). Darkly-stained specimens that were likely subject to prolonged periods of inundation did not preserve collagen; in bone samples lighter in color, collagen preservation was poor as well. We therefore turned to ^14^C AMS dating of the carbonate component in bioapatite in bone and enamel. Few publications have reported ^14^C dates on tooth enamel and bone bioapatite, as the method is destructive and less well established than that utilizing bone collagen as a source of radiocarbon. In pre-treatment of enamel, we used a vacuum as an alternative to acetic acid [44].

Our sampling strategy for human enamel involved replacing all sampled crowns with exact replicas in their original position. We first took photographs in multiple views of the teeth to be sampled. A silicone mold was made for each crown, from which we made a urethane cast. That cast was fixed in the position of the original crown, using the photographs of the original tooth row prior to sampling. Using this sampling protocol, we retained all information for subsequent dental anthropological analysis.

General concordance between dates on human burials with similar taphonomic signatures and buried in close proximity suggest that the apatite dating is yielding accurate results and that the burials represent a contemporaneous early Holocene cemetery. Five individuals dated to a narrow range of about 250 years (8640–8330 BP; Table 2, dates 1–7).

To test the reliability of using the carbonate component in bioapatite as a source of radiocarbon, we took multiple samples using different source materials from the same individual or feature (Table 6). The general concordance of dates validates the method, as seen in three dates from an early Holocene skeleton (Table 6, dates 1–3). The enamel sample, the crystalline structure of which makes isotopic exchanges more difficult, and the core of the femur, which is especially porous after hydrolysis of collagen, still provide concordant dates. This demonstrates the near absence of isotopic exchange under arid conditions. The third estimate of 8220±40 BP suggests slightly greater sensitivity to exchange in bone compared to enamel as expected.

**Table 6:** Convergence of multiple ^14^C AMS dates based on bioapatite radiocarbon from different materials from the same individual.

No.	Material Sampled	^14^C AMS Date
***Burial G3B8***		
**1**	enamel	8420±40 BP
**2**	bone (femur interior)	8470±40 BP
**3**	bone (femur surface)	8220±40 BP
***Burial G1B11***		
**4**	enamel	5940±40 BP
**5**	enamel	5620±40 BP
**6**	bone (femur)	5570±40 BP
***Burial G1B8***		
**7**	enamel	4590±40 BP
**8**	bone (femur)	4090±40 BP
***Bos taurus GF10***		
**9**	enamel	4990±40 BP
**10**	bone (mandible)	4930±40 BP

A second multiply-sampled individual (G1B11) is mid-Holocene in age (Table 6, dates 4–6). The discrepancy between dates from this individual is slightly greater and may be due to insufficient purification during pre-treatment. The enamel for one of these samples may have been imperfectly separated from the dentine. Under arid conditions, carbon isotope exchange almost always involves rejuvenation, during which total inorganic dissolved carbon (TIDC) from surface waters tends toward equilibrium with atmospheric CO_2_,. This process results in a younger date. Excluding other complicating factors, the older date (5940±40 BP) is more likely to be the most accurate.

A third human skeleton was sampled twice, using enamel and bone, and also yielded concordant results (Table 6, dates 7, 8). A concordant pair of dates was also obtained using enamel and bone from a cattle mandible (*Bos taurus*, GF10), which was found deflated on the surface of the paleolake deposit (Table 6, dates 9, 10).

The differences that are apparent between enamel and bone samples from the same individual suggests that bone shows some isotopic exchange with the TIDC surface waters. This rejuvenation results in an average decrease in age of 250 years, a minor difference for dates in the early and mid-Holocene. The isotopic exchange of carbon, in addition, does not seem to be linked to time (e.g. 8420 and 8470 BP), but rather to local variation in inundation or sediment. The δ^13^C from bones that experienced early diagenesis remains slightly more positive (e.g. G1B8), but this criterion is too tenuous to be reliable.

Convergence of dates based on multiple sources was also used to evaluate the age of features such as the middens or lakebed fauna. Clam shell from middens or the lakebed that was sampled in multiple ways (leached; chalky exterior shell; dense opalescent, unaltered inner shell) yielded generally concordant results.

In conclusion, dating of the bone and enamel bioapatite has provided the backbone for our chronology (Figure 2). If significant diagenesis were a confounding factor, the dates would show greater dispersion.

### Comparative Craniometric Analysis

Craniometric measurements were selected to provide maximum correspondence with previous work on North African Late Pleistocene and Holocene human remains [18], [26], [27]. Seven chronospatial sample units were defined in accordance with published radiometric dates and allocation of specific sites to particular North African cultures (Table 3, numbers 1–7). The principal components analysis includes all individuals for which data were available and the maximum number of craniometric variables. After individuals were allocated to one of the sampling units, variable means were calculated from the raw data for the following variables: LGO, BPX, LBN, HBB, AFR, APA, AOC, CFR, COC, HNP, HNZ, BNZ, BZY, BFW, BFX, HORC (Table 4). The resulting matrix of means was subjected to principal components analysis to capture the correlations among variable means in a more manageable number of dimensions (Figure 6). The strength of this approach is that it allows maximum inclusion of individuals and variables and thus captures the greatest possible genotypic structure. This approach, however, does not consider within-sample variability, and population affinity is based solely on mean differences.

Three of the loadings from the principal components analysis returned eigenvalues greater than 1.0 (Table 7). Factor loadings transparently reflect anatomical or functional units. The first principal component appears to most closely approximate a size vector, but the loadings are not uniformly positive. The pattern of positive and negative loadings for principal components 2 and 3 are not interpretable.

**Table 7:** Principal component loadings for human samples extracted from matrix of means for North African samples.

Measurement Acronym	PC1	PC2	PC3
**LGO**	0.963	0.129	0.227
**BPX**	0.873	0.463	−0.140
**LBN**	0.792	−0.055	0.392
**HBB**	0.704	−0.607	0.167
**AFR**	0.901	−0.373	−0.010
**APA**	−0.210	0.403	0.885
**AOC**	0.497	0.850	−0.169
**CFR**	0.857	−0.469	−0.098
**COC**	0.609	0.678	−0.182
**HNP**	0.763	−0.089	−0.327
**HNZ**	−0.289	0.445	−0.025
**BNZ**	−0.209	−0.114	0.916
**BZY**	0.496	−0.748	0.027
**BFW**	0.853	−0.433	0.256
**BFX**	0.418	0.842	0.222
**HORC**	0.760	0.645	0.077
**Eigenvalue**	7.455	4.396	2.174
**Percent Variance**	46%	27%	13%

Measurement abbreviations as in Table 4.

### Strontium Isotope Analysis

In order to investigate residential mobility and sedentism at the Gobero site complex, strontium isotope analysis was performed on archaeological human tooth enamel as well as modern baseline soil and faunal samples. At the Archaeological Chemistry Laboratory at Arizona State University (ASU), the teeth and bone samples were first mechanically cleaned by abrasion with a Dremel 3956-02 Variable Speed MultiPro drill equipped with an engraving cutter to remove the outermost layers of tooth enamel, which are most susceptible to diagenetic contamination. Eight milligrams of tooth enamel powder were dissolved in 0.50 mL of twice-distilled 5 M HNO_3_. Bone and soil samples were ashed at 800°C for 10 hours, and then soil samples were digested in concentrated HF and HNO_3_.

The strontium, uranium and neodymium concentrations were separated from the sample matrix in the W.M. Keck Foundation Laboratory for Environmental Biogeochemistry (ASU). The strontium was separated using EiChrom SrSpec resin, a crown-ether Sr-selective resin (100–15 µm diameter) loaded into the tip of a glass column. The SrSpec resin was pre-soaked and flushed with H_2_O to remove strontium present from the resin manufacturing process. The resin was further cleaned in the column with repeated washes of deionized H_2_O and conditioned with 750 µL of HNO_3_. The dissolved sample was loaded in 250 µL of 5 M HNO_3_, washed in 500 µL of 5 M HNO_3_, and then the strontium was eluted with 1000 µL of H_2_O. Samples were analyzed using the Neptune multi-collector inductively coupled plasma mass spectrometer (MC-ICP-MS). Recent ^87^Sr/^86^Sr analyses of strontium carbonate standard SRM-987 yield a value of ^87^Sr/^86^Sr = 0.710261±0.000020 (2σ), which is in agreement with analyses of SRM-987 using a thermal ionization mass spectrometer (TIMS), where ^87^Sr/^86^Sr = 0.710263±0.000016 (2σ) [45].

Uranium and neodymium concentrations were obtained on a quadrupole inductively-coupled plasma mass spectrometer (Q-ICP-MS) to monitor diagenetic contamination in the samples. Since biogenic enamel and bone should have very low concentrations of these elements, elevated concentrations of uranium and neodymium can be used as proxies for the presence of diagenetic contamination of trace elements such as strontium. The mean U/Ca and Nd/Ca ratios are low and within the range of published uncontaminated U/Ca ratios from enamel samples (U/Ca = 1.08×10^−6^±1.33×10^−6^, n = 7, 1σ; Nd/Ca = 3.73×10^−7^±3.94×10^−7^, n = 7, 1σ).

Baseline strontium isotope data from soil samples soil samples colleccted from burials show that ^87^Sr/^86^Sr = 0.71290±0.00064 (n = 7, 1σ), and modern faunal samples exhibit ^87^Sr/^86^Sr = 0.71261±0.00116 (n = 21, 1σ) (Table 8). The area around Gobero consists of Cretaceous sandstones that are likely to exhibit ^87^Sr/^86^Sr = 0.709–0.710, based on strontium isotope signatures from similar bedrock and archaeological human remains in Egypt and Libya [46], [47].

**Table 8:** Strontium isotope (^87^Sr/^86^Sr) results from human enamel in burials at Gobero and baseline samples from modern fauna and soil.

Laboratory Number	Burial or Specimen Number	Material (acronyms identify tooth sampled)	^87^Sr/^86^Sr
ACL-0305	G1B2	ULM1	0.71222
ACL-0306	G1B2	LRM2	0.71220
ACL-0307	G3B3	RC	0.71160
ACL-0308	G1B5	ULM2	0.71293
ACL-0314	G3B1	LLc	0.71143
ACL-0340	G1B11	URM1	0.71123
ACL-0341	G1B11	URM2	0.71147
ACL-0342	G1B11	URM3	0.71153
ACL-0442	G1B7	LRM3	0.71171
ACL-0443	G3B5	LLM2	0.71202
ACL-0444	G3B5	LLM1	0.71235
ACL-0445	G3B5	LLM3	0.71193
ACL-0446	G3B23	LLM1	0.71197
ACL-0447	G3B23	LLM2	0.71174
ACL-0448	G3B23	LLM3	0.71182
ACL-0449	G3B24	LLM1	0.71204
ACL-0450	G3B24	LLM2	0.71204
ACL-0451	G3B24	LLM3	0.71217
ACL-0452	G3B28	ULM1	0.71184
ACL-0453	G3B28	ULM2	0.71266
ACL-0454	G3B36	LLM1	0.71221
ACL-0456	G3B36	LLM3	0.71156
ACL-0457	G3B9	ULM1	0.71180
ACL-0458	G3B9	ULM2	0.71188
ACL-0459	G3B9	ULM3	0.71214
ACL-0460	G3B41	LLM1	0.71151
ACL-0461	G3B41	LLM2	0.71142
ACL-0462	G3B41	LLM3	0.71146
ACL-0545	GOB-BCS1	bone (*Capra hircus*)	0.71470
ACL-0546	GOB-BCS2	enamel (*Camelus dromedarius*)	0.71308
ACL-0547	GOB-BCS3	enamel (*Camelus dromedarius*)	0.71225
ACL-0548	GOB-BCS4	enamel (*Antilopini* sp.)	0.71157
ACL-0549	GOB-BCS5	bone (rodent)	0.71152
ACL-0550	GOB-BCS6	bone (rodent from *Bubo* sp. pellet)	0.71261
ACL-0551	GOB-BCS7	bone (rodent from *Bubo* sp. pellet)	0.71217
ACL-0552	GOB-BCS8	bone (rodent from *Bubo* sp. pellet)	0.71238
ACL-0553	GOB-BCS9	bone (rodent from *Bubo* sp. pellet)	0.71444
ACL-0554	GOB-BCS10	bone (rodent from *Bubo* sp. pellet)	0.71160
ACL-0555	GOB-BCS11	bone (rodent from *Bubo* sp. pellet)	0.71477
ACL-0556	GOB-BCS12	bone (rodent from *Bubo* sp. pellet)	0.71080
ACL-0557	GOB-BCS13	bone (*Antilopini* sp.)	0.71354
ACL-0558	GOB-BCS14	bone (caprine or ovine)	0.71371
ACL-0559	GOB-BCS15	bone (caprine or ovine)	0.71176
ACL-0560	GOB-BCS16	shell (snail)	0.71120
ACL-0561	GOB-BCS17	enamel (*Hippopotamus* sp.)	0.71122
ACL-0562	GOB-BCS18	bone (*Antilopini* sp.)	0.71251
ACL-0563	GOB-BCS19	bone (*Antilopini* sp.)	0.71252
ACL-0566	GOB-BCS2	enamel (*Camelus dromedarius*)	0.71306
ACL-0567	GOB-BCS2	enamel (*Camelus dromedarius*)	0.71334
ACL-0643	GOB-SOI-03	soil (from burial G1B17)	0.71326
ACL-0644	GOB-SOI-04	soil (from burial G1B18)	0.71347
ACL-0645	GOB-SOI-05	soil (from burial G1B6)	0.71384
ACL-0655	GOB-SOI-16	soil (from burial G3B23)	0.71258
ACL-0660	GOB-SOI-21	soil (from burial G3B4)	0.71245
ACL-0665	GOB-SOI-26	soil (from burial G3B7)	0.71204
ACL-0667	GOB-SOI-28	soil (from burial G3B9)	0.71269

*Abbreviations*: *ACL*, Archaeological Chemistry Laboratory, Arizona State University; *c*, deciduous canine; *L*, lower (or left); *M*, molar; *R*, right; *U*, upper.

Our preliminary archaeological human enamel data from Gobero (Table 8, ^87^Sr/^86^Sr = 0.71189±0.00039, n = 28, 1σ) closely matches the baseline data outlined above, which suggests that the sampled individuals were not moving between, or migrating from, areas with much lower strontium isotope signatures, such as the Cenozoic volcanic terrain of the Aïr highlands in northern Niger and Hoggar in southern Algeria (^87^Sr/^86^Sr = 0.703) [48]–[51].

### Pollen Analysis

A total of 59 pollen samples were collected from 23 burials (Table 9). Pollen samples were prepared using 10% Na-pyrophosphate, sieving using 200–300 µm and 7 µm meshes, 10% HCl, acetolysis, heavy liquid separation (Na metatungstate hydrate with specific gravity of 2.0 and centrifugation at 2000 rpm for 20 minutes), 40% HF, and mounting with glycerol jelly on permanent slides. *Lycopodium* spores were added to calculate pollen concentration (pollen grains per gram = p/g), and pollen analyses were performed at 400× and 1000× with immersion oil for critical determinations. Graphs are drawn with Tilia and TGView [52], and results are presented using pollen sums of 300–400 grains (Figure 10).

**Table 9:** Pollen sampling for three mid-Holocene burials.

Sample No.	Description
***G1B18*** * (not dated)*	
**1**	under the skull
**2**	under the ribs
**3**	inside the stomach
**4**	near and inside left hand
**5**	under the ribs after removal of skeleton
***G1B8-10*** * (2860–2490 cal BC) (triple burial, * *Figure 5E, F* *)*	
**6**	behind thorax of adult G1B8
**7**	between ribs and pelvis to the left of adult G1B8
**8**	between arms of juveniles G1B9 and G1B10
***G3B36*** * (3630–3370 cal BC) (male with vessel, * *Figure 7A* *)*	
**9**	under the skull
**10**	under the femur
**11**	near warthog tusk
**12**	between left arm and ribs
**13**	inside pot containing skull

Preliminary data on early Holocene pollen were obtained from seven samples from three burials on site G3 (G3B17, G3B8, G3B9). Concentrations are less than 500 p/g, and two of the samples are sterile. Most pollen is crumpled. Poaceae comprise half of the spectra with a significant amount of Cyperaceae and *Cornulaca*. Arboreal pollen includes *Ficus*, *Tamarix* and *Myrtus*. Aquatics include *Juncus*, *Nymphaea* and *Potamogeton*, and many hyphas and fungal spores were observed in G3B17 and G3B8. Microscopic charcoal is present (>125 µm) in burial in G3B8, suggesting the presence of a hearth in the vicinity of the burial.

Mid-Holocene pollen was analyzed on the basis of 13 samples from burials at sites G1 and G3 (Table 9). Pollen is present in all samples, although concentrations are very low, frequently less than 300 p/g and always less than 600 p/g. The diversity of pollen taxa per sample are also low (minimum 14, maximum 32). When samples from a single burial are combined, diversity remains low in burial G1B18 (53 taxa) but increases in the other two burials (68–78 taxa). Pollen clusters of several taxa are present in sample 3a between the arms of the triple burial (Table 9). Pollen spectra are herb-dominated with prevalence of Poaceae (ca. 60% on average). Cyperaceae and *Cornulaca* are common with significant percentages in many samples. *Cornulaca*, for example, is greater than 30% in samples 3a (G1B8-10) and 4b and 5b (G1B18). The ratio D-desert / Sh-Sahelian elements was less than 1.0, the only exception being sample 4b from burial G1B18. Arboreal pollen, which is always less than 5% of total pollen, includes the ubiquitous Sudanian *Ficus* plus Moraceae undiff. and the Sahelian *Salvadora* and *Ziziphus* (Figure 10B). The Sahelian *Capparis* is present in burials G1B18 and G3B36. Desert trees such as *Acacia* are rare, occurring only in sample 1a of the triple burial G1B8-10. Sudanian alluvial or riverine vegetation, such as *Celtis*, *Ficus*, *Salvadora persica* and *Hyphaene thebaica*, is more common in G1B8-10 (1.5%) than in the other two burials (0.5–0.8%). Saharo-Sindian shrubs and herbs, such as *Cornulaca*, Cichoriae undiff., *Ephedra*, *Plantago*, *Heliotropium*, *Zygophyllum*, are widespread in the spectra. Pioneer elements, such as Compositae, *Polycarpaea* and *Plantago*, are present especially in burial G1B8-10. *Boerhavia*-type pollen that characterizes a semidesert environment is significant (3.1%) in sample 1b from burial G1B18. *Celosia*-type *trygina* (wool flower) was unusually common (32.4%) in sample 3a from burial G1B8-10, where it occurred in clusters.

Herb plants of wet environments, which measured 0.6–0.7% excluding Cyperaceae, were found in the majority of samples and were slightly more common in burial G3B36. The hydrophytes *Lemna* and *Potamogeton*, both of which have submerged and floating leaves, were present in G1B18 and G3B36 (Figure 10C). *Typha* (cat tail) was found only in trace amounts in sample 2c (G3B36). Algal elements were observed in G1B8-10 and G1B18 (here also including *Concentricystes* which are algal spores of Zignemataceae) [53]. On the other hand, graminoid-type phytolits, which are silicate or ossalate inclusions of plant epidermids, were abundantly observed in sample 1b from burial G1B18.

Pollen data from the burials are fairly concordant for interpreting the Gobero plant landscape, which was largely dominated by an open grass savanna [15]. Sahelian elements (Poaceae+Cyperaceae) predominate over desert vegetation (Chenopodiaceae+Asteraceae). Under conditions of increasing aridity and near salty environments, the Sahelian savanna shifted toward a Saharan environment, with increasing Poaceae and Chenopodiaceae including alophylous shrubs largely spread in salt-rich soils. Chenopodiaceae are common and associated with Poaceae and Cyperaceae. On the other hand, several pollen and other sporomorphs suggest wet environments. For example, Cyperaceae, which includes both aquatic and dune herb species, may result from sedge belts in the vicinity of a lake, and hydrophytes and algal elements thrive in permanent fresh water. The hygrophilous fig tree (*Ficus*) probably had a scattered distribution near the wettest places of the area, which it may have shared with *Capparis*, *Ziziphus*, *Salvadora* and *Cadaba*.

Sudanian and tropical elements, such as *Celtis*, *Ficus*, *Salvadora persica*, *Hyphaene thebaica*, could have been part of riverine forests that also included *Combretum*, *Cadaba*, *Ziziphus*, *Pterocarpus* and *Capparis*. None of these taxa, however, occur in very high frequencies among non-arboreal pollen (Figure 10C). Pollen from burial samples, in sum, suggests that the landscape was a mosaic of xeric and wet environments.
